# Supplementary material for: The Role of Extracellular Vesicles in Ischemic Stroke Severity
Source: Biology (Basel). 2022 Oct 11;11(10):1489. doi: 10.3390/biology11101489 (PMC9598264; doi:10.3390/biology11101489)
Supplement: Supplementary file 1 [file biology-11-01489-s001.zip › biology-1932341-supplementary.pdf]

Supplementary Table S1
MISEV 2018 guidelines compliance

	Section title	Required information according to MISEV2018	Mandatory requirement	Not applicable/not available	Our approach	Compliance with MISEV2018 requirements
1	Nomenclature	The term extracellular vesicle (EV) can be used with demonstration of extracellular (no intact cells) and vesicular nature per these characterization and function	YES		As explained in section 4 and 5, the term extracellular vesicle (EV) has been used in the manuscript	YES
2a	Collection and pre-processing (tissue culture conditioned medium)	General cell characterization	YES	N/A		
2a	Collection and pre-processing (tissue culture conditioned medium)	Medium used before and during collection (additives, serum, other)	YES	N/A		
2a	Collection and pre-processing (tissue culture conditioned medium)	Exact protocol for depletion of EVs from additives in collection medium	YES	N/A		
2a	Collection and pre-processing (tissue culture conditioned medium)	Nature and size of culture vessels, and volume of medium during conditioning<br>e) specific culture conditions (treatment, % O2, coating,polarization…) before and during collection	N/A			
2a	Collection and pre-processing (tissue culture conditioned medium)	Number of cells/ml and % of live/ dead cells at time of collection	N/A			
2a	Collection and pre-processing (tissue culture conditioned medium)	Frequency and interval of Conditioned Medium harvest	N/A			
2b and 2c	Collection and pre-processing (Biofluids or tissues)	Donor status if available (age, sex, food/water intake, collection time, disease, medication, other)	YES		Clinical records were collected for each subject<br>Blood samples were processed within 2 hrs after the blood draw	YES
2b and 2c	Collection and pre-processing (Biofluids or tissues)	Volume of biofluid or volume/mass of tissue sample collected per donor	YES		For each subject, two blood samples were collected in an EDTA tube (7 mL)	YES
2b and 2c	Collection and pre-processing (Biofluids or tissues)	Total volume/mass used for EV isolation (if pooled from several donors)	YES		EVs isolation was performed starting from 1.5 mL of plasma. Each EV pellet aliquot obtained from	YES

					each subject was resuspended in 500 µl of PBS triple filtered (pore size 0.1 µm).	
2b and 2c	Collection and pre-processing (Biofluids or tissues)	All known collection conditions, including additives, at time of collection			Blood samples were collected in 7 mL EDTA tubes	YES
2b and 2c	Collection and pre-processing (Biofluids or tissues)	Pre-treatment to separate major fluid-specific contaminants before EV isolation			Blood was centrifuged at 1200 × *g* for 15 min at room temperature. 1.5 ml of plasma were further centrifuged at 1000, 2000, and 3000 × *g* for 15 min at 4 °C. The obtained pellets were discarded to remove cell debris.	YES
2b and 2c	Collection and pre-processing (Biofluids or tissues)	Temperature and time of biofluid/tissue handling before and during pre-treatment			Each sample was processed within 2 hrs from blood draw.	YES
2b and 2c	Collection and pre-processing (Biofluids or tissues)	For cultured tissue explants: volume, nature of medium and time of culture before collecting conditioned medium		N/A		
2b and 2c	Collection and pre-processing (Biofluids or tissues)	For direct tissue EV extraction: treatment of tissue to release vesicles without disrupting cells		N/A		
2d	Storage and recovery	Storage and recovery (e.g., thawing) of CCM, biofluid, or tissue before EV isolation (storage temperature, vessel, time; method of thawing or other sample preparation)	N/A			
2d	Storage and recovery	Storage and recovery of EVs after isolation (temperature, vessel, time, additive(s)…)	YES		Isolated EVs were immediately characterized by flow cytometry and Nanosight.	YES
3	EV separation and concentration	Centrifugation: reference number of tube(s), rotor(s), adjusted k factor(s) of each centrifugation step (= time+speed+ rotor, volume/density of centrifugation conditions), temperature, brake settings	YES		Plasma was centrifuged at 1000, 2000, and 3000 × g for 15 min at 4 °C (Haereus Labofuge 400R, Hanau, Germany). EVs were then isolated from supernatants by ultracentrifugation at 110,000 × g for 75 min at 4 °C in polypropylene	YES

				ultracentrifuge tubes (Quick-Seal, Round-Top, polypropylene, 13.5 mL; Beckman Coulter, Inc., Indianapolis, IN, USA) rotor MLA-55 (Beckman Coulter), filled with PBS previously filtered through a 0.10-µm pore-size polyethersulfone filter (StericupRVP, Merck Millipore; Burlington, MA, USA). Our method is included in the category "Intermediate recovery, intermediate specificity = mixed EVs with limited non-EV components"
3	EV separation and concentration	Density gradient: nature of matrix, method of generating gradient, reference (and size) of tubes, centrifugation speed and time (with brake specified), method and volume of fraction recovery.	N/A	
3	EV separation and concentration	Chromatography: matrix (nature, pore size,…), loaded sample volume, fraction volume, number	N/A	
3	EV separation and concentration	Precipitation: reference of polymer, ratio vol/vol or weight/vol polymer/fluid, time/temperature of incubation, time/speed/temperature of centrifugation	N/A	
3	EV separation and concentration	Filtration: reference of filter type (=nature of membrane, pore size…), time and speed of centrifugation, volume before/after (in case of concentration)	N/A	
3	EV separation and concentration	Antibody-based : reference of antibodies, mass Ab/amount of EVs, nature of Ab carrier (bead, surface) and amount of Ab/carrier surface	N/A	
3	EV separation and concentration	Other: all necessary details to allow replication	N/A	

3	EV separation and concentration	Additional step(s) to concentrate, if any		N/A		
3	EV separation and concentration	Additional step(s) to wash matrix and/or sample, if any		N/A		
4a	EV characterization, Quantification	Volume of fluid, and/or cell number, and/or tissue mass used to isolate EVs	N/A			
4a	EV characterization, Quantification	Global quantification by at least 2 methods: protein amount, particle number, lipid amount, expressed per volume of initial fluid or number of producing cells/mass of tissue	YES		To quantify the total number of EVs we applied the two following approaches:<br>1) Nanoparticle tracking analysis by NanoSight LM10-HS system (NanoSight Ltd., Amesbury, UK) Instrument specifications:<br>camera type: sCMOS<br>laser: 488nm (blue)<br>gain settings: 1<br>camera Shutter: 20 ms<br>histogram upper and lower limits: 1000nm and 1nm respectively<br>frame rate: 24.99<br>temperature: 20-28°C<br>syringe pump speed: 30<br>detection threshold: 10<br>viscosity: 0.86cP-0.91cP<br><br>Five 30-s recordings were made for each sample. Collected data were analyzed with NTA software (Malvern Panalytical Ltd.), which provided high-resolution particle-size distribution profiles as well as measurements of the EV concentration.<br>2) High-resolution Flow cytometry by MACSQuant Analyzer 10, Miltenyi Biotec. To analyze EV integrity, 60 µl aliquots were stained with 0.02 µM 5(6)-carboxyfluorescein diacetate N-succinimidyl ester	YES

				(CFSE) at 37 °C for 20 min in the dark.	
4a	EV characterization, Quantification	Ratio of the 2 quantification figures	YES	N/A	
4b	EV characterization, General Characterization	At least <u>three</u> positive protein markers of EVs, including at least one transmembrane/lipid bound protein and one cytosolic protein<br><br>At least one negative protein marker	YES	The following antibodies have been used to measure positive protein markers of plasmatic EVs: AbCD14-APC (monocytes, clone TUK4, Miltenyi Biotec), AbCD61-APC (platelets, clone Y2/51, Miltenyi Biotec), AbCD62E-APC (activated epithelium, clone REA280, Miltenyi Biotec), Ab CD105-APC (clone: 43A4E1, Miltenyi Biotec), AbCD25-APC (clone: 4E3, Miltenyi Biotec). CFSE staining was used to assess cytosolic esterase activity. As negative control, representative EVs samples were incubated with Tween20 (P1379; Merk, Sigma-Aldrich) ON at 37 °C, and then incubated with CFSE as described above.	YES
4c	Single EV characterization	Images of single EVs by electron microscopy	YES	Transmission Electron Microscopy (TEM) analysis was performed on random samples as quality control.	YES
4c	Single EV characterization	Non-image-based method analysing large numbers of single EVs: Non-image-based method analysing large numbers of single EVs: NTA, TRPS, FCS, high-resolution flow cytometry, multi-angle light-scattering, Raman spectroscopy, etc.	YES	NTA and Flow Cytometry were performed (see above).	YES
5	Functional studies	Dose-response assessment	N/A		
5	Functional studies	Negative control = nonconditioned medium, biofluid/tissue from control donors, as applicable	N/A		

5	Functional studies	Quantitative comparison of functional activity of total fluid, vs EV-depleted fluid, vs EVs (after high recovery/low specificity separation)	N/A	
5	Functional studies	Quantitative comparison of functional activity of EVs vs other EPs/fractions after low recovery/high specificity separation	N/A	
5	Functional studies	Quantitative comparison of activity of EV subtypes (if subtype-specific function claimed)	N/A	
5	Functional studies	Extent of functional activity in the absence of contact between EV donor and EV recipient	N/A	
6	Reporting	Submission of data (proteomic, sequencing, other) to relevant public, curated databases or open-access repository	YES	N/A

Supplementary Table S2. Relationships between outcomes of stroke severity and hypertension and smoke. Negative binomial regression models were adjusted by age, gender, BMI, glucose level and therapy only for NIHSS after one week and mRS, we reported geometric marginal means.

	NIHSS T0				NIHSS Tw				mRS after 3 months			
**Hypertension**	**Mean**	**95% CI**		**P-value**	**Mean**	**95% CI**		**P-value**	**Mean**	**95% CI**		**P-value**
Yes	11,8	7,4	18,8	0,3023	**11,0**	**4,7**	**25,6**	**0,0411**	1,5	0,6	4,3	0,2694
No	9,1	7,5	11,1		**4,0**	**2,5**	**6,2**		2,8	2,0	4,0	
**Smoke**	**Mean**	**95% CI**		**P-value**	**Mean**	**95% CI**		**P-value**	**Mean**	**95% CI**		**P-value**
Yes	12,3	8,3	18,3	0,0784	7,2	3,6	14,5	0,6442	2,3	1,0	4,9	0,6737
No	8,7	6,9	10,9		6,1	3,9	9,5		1,9	1,2	3,2	

Abbreviations: NIHSS, National Institutes of Health Stroke Scale; mRS, Modified Rankin Scale.

Supplementary Table S3. Quantification of total EVs and EV subtypes.

		**T0 (N=47)** **Median [Q1; Q3]**	**T1 (N=33)** **Median [Q1; Q3]**	**Tweek (N=18)** **Median [Q1; Q3]**
**EV count**[a] (x10^6/ml plasma)	**Total EVs**	4201 [2913 ; 5855]	3196 [2556 ; 4723]	4300 [2803 ; 6444]
**EV subtype**[b] (x10^3/ml plasma)	**CD14+** (macrophages/monocytes)	16 [8 ; 24]	12 [5 ; 28]	18 [13 ; 25]
	**CD61+** (platelets)	61 [25 ; 158]	69 [24 ; 143]	54 [41 ; 75]
	**CD105+** (endothelium)	13 [6 ; 19]	13 [5 ; 25]	18 [10 ; 20]
	**CD25+** *(T-cells)*	9 [4 ; 14]	8 [4 ; 16]	9 [6 ; 12]
	**CD62E+** *(activated endothelial cells)*	14 [8 ; 22]	16 [6 ; 35]	20 [14 ; 25]

a Counts of total EVs obtained by Nanosight analysis.
b Counts of EV subtypes obtained by flow cytometry.
Abbreviations: EV, extracellular vesicles.

Supplementary Table S4. Relationships between Total EV concentrations and hypertension and smoke. Negative binomial regression models were adjusted by age, gender, BMI, glucose level. We reported geometric marginal means.

	**EV total (count/ml PL)**			
**Hypertension**	**Mean**	**95% CI**		**P-value**
Yes	4734500000	2865100000	7823400000	0,7405
No	5177400000	4156100000	6449700000	
**Smoke**	**Mean**	**95% CI**		**P-value**
Yes	4935100000	3203700000	7602200000	0,9765
No	4966900000	3846900000	6413000000	

Abbreviations: EV, extracellular vesicles.

Supplementary Table S5. Relationships between EV subtypes and hypertension and smoke. Negative binomial regression models were adjusted by age, gender, BMI, glucose level. We reported geometric marginal means.

	**CD14+ (count/ml PL)**				**CD61+ (count/ml PL)**				**CD105+ (count/ml PL)**				**CD25+ (count/ml PL)**				**CD62E+ (count/ml PL)**			
**Hypertension**	**Mean**	**95% CI**		**P-value**	**Mean**	**95% CI**		**P-value**	**Mean**	**95% CI**		**P-value**	**Mean**	**95% CI**		**P-value**	**Mean**	**95% CI**		**P-value**
Yes	19332	6904	54127	0,8481	237810	96739	584597	0,0580	**27278**	**12746**	**58379**	**0,0352**	13084	6534	26198	0,2656	24088	11046	52528	0,4158
No	21457	14175	32479		92919	61429	140552		**11425**	**8529**	**15306**		8648	6404	11679		17124	12597	23278	
**Smoke**	**Mean**	**95% CI**		**P-value**	**Mean**	**95% CI**		**P-value**	**Mean**	**95% CI**		**P-value**	**Mean**	**95% CI**		**P-value**	**Mean**	**95% CI**		**P-value**
Yes	14467	5928	35307	0,1233	139580	63959	304614	0,7518	17042	9338	31101	0,8067	9254	5086	16836	0,3559	22156	11828	41502	0,5737
No	28672	17481	47025		158310	100789	248660		18287	12679	26377		12227	8605	17374		18617	12571	27571	

Abbreviations: CD14+, macrophages/monocytes; CD61+, platelets; CD105+, endothelium; CD25+, T-cells; CD62E+, activated endothelial cells.
